# CD40LG/CD28-Mediated Rho GTPase Signaling Drives Survival and Chemoresistance in Non-ETP T-ALL

**DOI:** 10.3390/ijms27125306

**Published:** 2026-06-11

**Authors:** Yan Yang, Wei Lu, Zhexi Zhu, Chenyang Li, Zihao Guo, Han Zhang

**Affiliations:** 1 Institute of Medical Biology, Chinese Academy of Medical Sciences & Peking Union Medical College, Kunming 650118, China; 2 Graduate School, Kunming Medical University, Kunming 650500, China

**Keywords:** T-cell acute lymphoblastic leukemia, non-early T-cell precursor acute lymphoblastic leukemia, CD40 ligand, CD28, PI3K, NF-κB, Rho GTPase

## Abstract

T-cell acute lymphoblastic leukemia (T-ALL) is an aggressive subtype of ALL characterized by unfavorable clinical outcomes. Despite significant progress in deciphering the genetic and epigenetic landscapes of T-ALL, the underlying molecular mechanisms, particularly in non-early T-cell precursor (non-ETP) T-ALL, remain incompletely understood. In this study, functional assays were performed using three well-characterized non-ETP T-ALL cell lines. In vivo therapeutic efficacy was evaluated using non-ETP T-ALL xenograft models. Transcriptomic profiling was performed by RNA sequencing (RNA-seq) followed by bioinformatic analysis. Publicly available clinical datasets from T-ALL patients were mined to analyze survival outcomes. We found that activation of CD40 ligand (CD40LG) or CD28 accelerates cell-cycle progression and enhances the migratory capacity of non-ETP T-ALL cells, with CD40LG uniquely upregulating CXCR4 to mediate bone marrow tropism. Further RNA-seq and functional validation identified Rho GTPase signaling, specifically RhoA/Rac1/Rac2, as a pivotal downstream effector of CD40LG/CD28, leading to therapeutic resistance to PI3K inhibition. Pharmacological blocking RhoA or Rac1 using small-molecule compounds not only induces remarkable cytotoxicity but also sensitizes resistant cells to PI3K inhibitors, both in vitro and in vivo. Clinically, elevated expression of *CD40LG*, *CD28*, *RHOA*, or *RAC2* correlates with poor prognosis in non-ETP T-ALL patients. These findings uncover a novel CD40LG/CD28-Rho GTPase axis as a key driver of pathogenesis and a potential therapeutic vulnerability in non-ETP T-ALL, providing a new target for precision intervention and a promising strategy to overcome therapeutic resistance.

## 1. Introduction

T-cell acute lymphoblastic leukemia (T-ALL) is a highly aggressive hematologic malignancy arising from the malignant transformation of T-cell progenitors, accounting for approximately 15% of pediatric and 25% of adult ALL cases [1,2,3]. Immunophenotypically, T-ALL is broadly stratified into early T-cell precursor ALL (ETP-ALL) and non-ETP T-ALL, with the former representing a high-risk subtype characterized by an extremely poor prognosis [4]. Despite advances in intensive multidrug chemotherapy regimens and robust supportive care, nearly 15% of pediatric and 50% of adult T-ALL patients experience chemoresistance, which often leads to disease relapse or treatment failure [2,5]. Crucially, although non-ETP T-ALL is generally associated with a better prognosis than ETP-ALL, a considerable proportion of patients progress to relapsed or refractory (R/R) disease [6,7]. This clinical discrepancy highlights the insufficient understanding of the molecular mechanisms driving the pathogenesis of non-ETP T-ALL. Although activating mutations in *NOTCH1* have been implicated in T-ALL development, γ-secretase inhibitors targeting this pathway have yielded limited clinical efficacy [8,9], highlighting the urgent need to explore novel mechanisms.

The CD40 ligand (CD40LG, also known as CD154/gp39) and CD28 are pivotal T-cell costimulatory molecules. CD40LG is restricted to activated mature thymocytes and diverse immune cells (e.g., mast cells, dendritic cells, natural killer cells) [10,11,12,13,14], where it binds CD40 to regulate adhesion, cytokine secretion, and tumor progression [15,16,17,18,19,20,21,22]. In addition to its function as a ligand for CD40, CD40LG also acts as a signaling molecule in T cells mediating T-cell activation [23,24], but its role in T-cell malignancies remains obscure. In contrast, CD28 expression begins on mature double-positive thymocytes and increases as maturation progresses [25]. Engagement of CD28 with CD80 (B7-1) and CD86 (B7-2) on antigen-presenting cells delivers essential activation signals for T-cell activation [26]. As such, activating genetic alterations in *CD28* have been identified across multiple T-cell malignancies, including T-cell leukemia/lymphoma (ATLL), peripheral T-cell lymphoma, cutaneous T-cell lymphoma, angioimmunoblastic T-cell lymphoma, and lymphomas derived from follicular T-helper cells [27,28,29,30]. CD28 overexpression also serves as an independent prognostic biomarker for unfavorable prognosis in ATLL patients [31]. Despite these associations, how CD28 drives T-cell malignancies is still unclear.

Interestingly, our prior work established that CD40LG and CD28 are indispensable for non-ETP T-ALL cell growth and survival, with CD40/CD40LG primarily governing bone marrow (BM) infiltration in xenograft models [32]. However, the downstream signaling cascades mediating these effects and their link to chemotherapeutic response remain undefined. Building on these findings, we further dissected CD40LG/CD28 downstream pathways in non-ETP T-ALL cells, revealing a Rho GTPase-dependent mechanism that drives survival and therapeutic resistance.

## 2. Results

### 2.1. CD40LG/CD28 Activation Promotes Non-ETP T-ALL Growth Primarily via Cell-Cycle Progression

To dissect the regulatory mechanisms underlying CD40LG/CD28 signaling in non-ETP T-ALL, we selected three human non-ETP T-ALL cell lines with distinct molecular heterogeneity: Jurkat (harboring mutations in *PTEN*, *TP53*, etc.) [33,34,35], MOLT-4 (harboring mutations in *TP53*, *PIC1*, etc.) [36,37] and CCRF-CEM (hereafter referred to as CEM, harboring mutations in *TP53*, *CCND1*, etc.) [35]. To specifically activate CD40LG and CD28 signaling, cells were treated with recombinant human CD40 (rhCD40) and agonistic anti-human CD28 monoclonal antibodies (hCD28-mAbs), respectively. Flow cytometry analysis showed that no obvious alterations in surface CD40LG expression were observed between the resting and stimulated groups (Supplementary Figure S1A). In contrast, nearly all cells (99.5–100%) were CD28-positive (CD28^+^) in both basal and stimulated states. Although the positive cell percentage was unchanged, the mean fluorescence intensity (MFI) of surface CD28 dropped markedly upon stimulation (Supplementary Figure S1B), indicating stimulation-triggered internalization of CD28.

**Figure 1:**
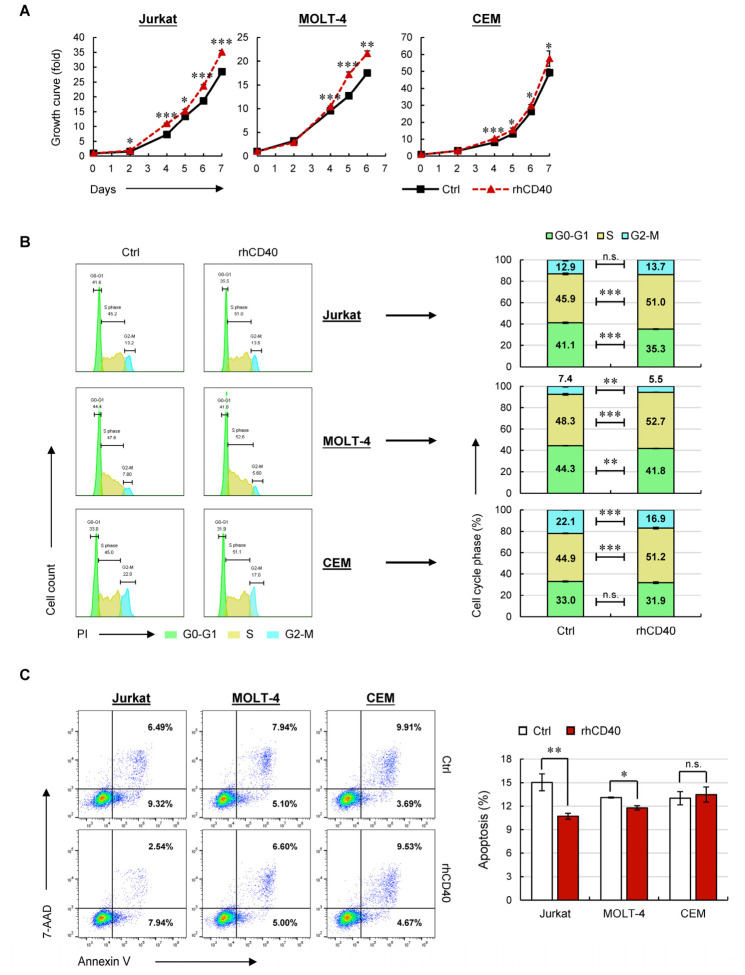
Activation of CD40LG signaling promotes non-ETP T-ALL growth primarily through cell-cycle progression. (**A**) Relative viable cell counts in Jurkat, MOLT-4 and CEM cells treated with vehicle control or rhCD40. (**B**) Representative cell-cycle distribution (left) and quantification of cell percentages in each phase (right) for Jurkat, MOLT-4 and CEM cells treated with vehicle control or rhCD40. Data is shown as mean ± SD. (**C**) Representative apoptosis (left) and quantification of Annexin V^+^ apoptotic cells (right) for Jurkat, MOLT-4 and CEM cells treated with vehicle control or rhCD40. Data is shown as mean ± SD. n.s., not significant, * *p* < 0.05; ** *p* < 0.01; *** *p* < 0.001.

**Figure 2:**
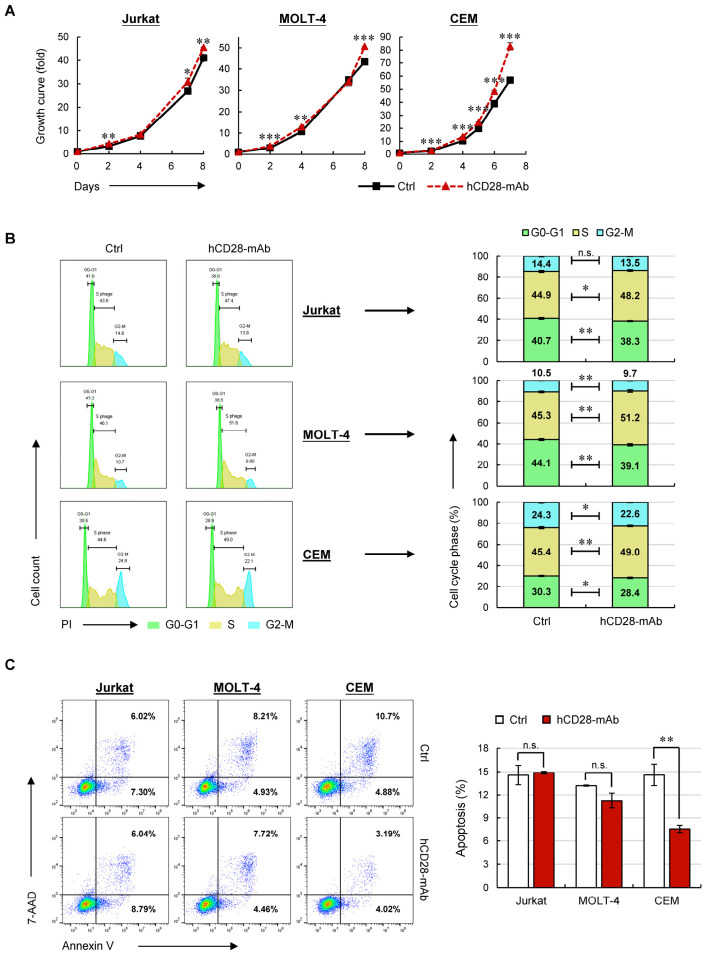
Activation of CD28 signaling promotes non-ETP T-ALL growth primarily through cell-cycle progression. (**A**) Relative viable cell counts in Jurkat, MOLT-4 and CEM cells treated with vehicle control or hCD28-mAb. (**B**) Representative cell-cycle distribution (left) and quantification of cell percentages in each phase (right) for Jurkat, MOLT-4 and CEM cells treated with vehicle control or hCD28-mAb. Data are shown as mean ± SD. (**C**) Representative apoptosis (left) and quantification of Annexin V^+^ apoptotic cells (right) for Jurkat, MOLT-4 and CEM cells treated with vehicle control or hCD28-mAb. Data are shown as mean ± SD. n.s., not significant, * *p* < 0.05; ** *p* < 0.01; *** *p* < 0.001.

Distinct from the inhibitory effect of CD40LG knockdown reported in our previous work [32], exogenous activation of CD40LG modestly promoted cell growth compared with controls (Figure 1A), a phenomenon likely constrained by the intrinsically high basal proliferative capacity of non-ETP T-ALL cells. Further cell-cycle analysis revealed a 9–14% increase in the proportion of S-phase cells upon CD40LG stimulation (Figure 1B), while apoptosis assays showed a survival advantage specifically in Jurkat and MOLT-4 cells (Figure 1C). These data suggest that CD40LG primarily facilitates cell proliferation by accelerating S-phase progression in non-ETP T-ALL cells, whereas its pro-survival function is modest and cell line-dependent. Consistently, CD28 activation also enhanced cell growth (Figure 2A), which was primarily mediated by S-phase cell accumulation (Figure 2B) and partial suppression of apoptosis in CEM cells (Figure 2C). Notably, combined stimulation with rhCD40 and hCD28-mAb substantially inhibited apoptosis across all three cell lines (Supplementary Figure S2), suggesting a potential synergistic crosstalk between the CD40LG and CD28 signaling pathways.

Collectively, CD40LG and CD28 share a common mechanism to boost cell growth by facilitating cell-cycle progression, whereas they exert distinct effects on the regulation of cell survival.

### 2.2. CD40LG Specifically Enhances BM Homing of Non-ETP T-ALL Cells via CXCR4 Upregulation

**Figure 3:**
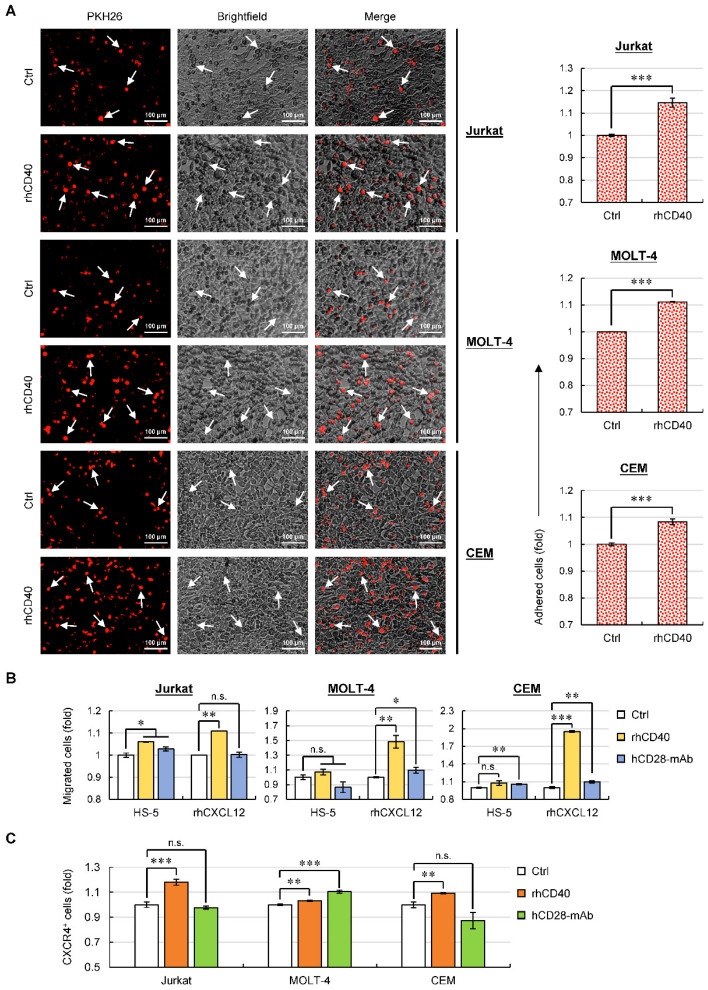
CD40LG enhances cell adhesion and BM homing of non-ETP T-ALL cells via CXCR4 upregulation. (**A**) Representative microscopic images of adherent Jurkat, MOLT-4 and CEM cells treated with vehicle control or rhCD40, with white arrows indicating leukemic cells adhering to BMSCs (left, scale bar, 100 μm). PKH26 fluorescence intensity in rhCD40-treated cells normalized to control (right). Data are shown as mean ± SD. (**B**) PKH26 fluorescence intensity of migrated cells normalized to control. Data are shown as the mean ± SD. (**C**) Flow cytometric analysis CXCR4 surface expression in rhCD40- or hCD28-mAb-treated cells, and percentage of CXCR4^+^ cells in the population is normalized to control. n.s., not significant, * *p* < 0.05; ** *p* < 0.01; *** *p* < 0.001.

Based on our prior observation that CD40LG or CD28 knockdown compromises adhesion of non-ETP T-ALL cells to bone marrow stromal cells (BMSCs) [32], we next questioned whether activation of these pathways reverses this adhesive impairment. Indeed, CD40LG activation markedly increased cell adhesion to HS-5 BMSCs relative to controls (Figure 3A), whereas CD28 stimulation had no such effect (Supplementary Figure S3), suggesting that CD28 molecules per se, rather than CD28-mediated signaling, may contribute to adhesion via unidentified mechanisms.

Next, we performed Transwell assays to model BM homing, with lower chambers containing culture media supplemented with HS-5 BMSCs or C-X-C motif chemokine ligand 12 (CXCL12). Interestingly, activation of CD40LG enhanced migration of all three cell lines toward the CXCL12-enriched microenvironment, and only Jurkat cells exhibited preferential migration toward the HS-5-containing setting. In contrast, CD28 stimulation elicited migration of selective cell lines to either the CXCL12- or HS-5-enriched microenvironments (Figure 3B). These results indicated that the pro-migratory effects of CD40LG or CD28 signaling on BM homing are predominantly mediated by CXCL12-dependent chemotaxis.

Given that stromal-vascular BM niches secrete abundant CXCL12 and that its receptor CXCR4 is highly expressed on leukemic cells, the CXCL12/CXCR4 axis is critical for BM homing [8]. We thus evaluated whether CD40LG/CD28 activation modulates CXCR4 expression of non-ETP T-ALL cells. As shown in Figure 3C, CD40LG activation upregulated surface CXCR4 expression in all cell lines, whereas CD28 stimulation exerted effects solely on MOLT-4 cells. Together, these data identified CD40LG as a key driver of BM tropism via CXCR4-mediated chemotaxis, a mechanism distinct from that of CD28.

### 2.3. Rho GTPase Signaling Emerges as a Pivotal Downstream Effector of CD40LG/CD28 Signaling

**Figure 4:**
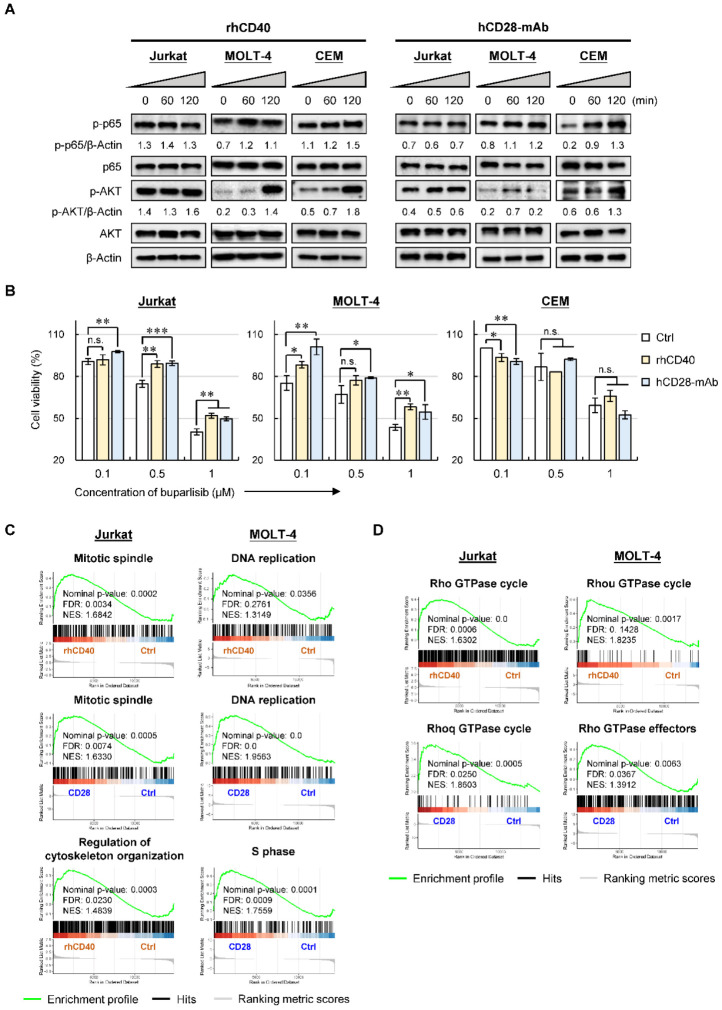
Downstream signaling pathways activated by CD40LG or CD28. (**A**) Immunoblots of p-p65, p65, p-AKT and AKT in cells stimulated with rhCD40 (left) or hCD28-mAb (right) at indicated time points, with β-Actin as a loading control. The p-p65/β-Actin and p-AKT/β-Actin values are indicated per lane. (**B**) Jurkat, MOLT-4 and CEM cells were treated with buparlisib in combination with vehicle control, rhCD40 or hCD28-mAb for 48 h. Cell viability was measured via CellTiter Blue assays, normalized to control. Data are shown as the mean ± SD. GSEA of pathways related to DNA replication and cell-cycle progression (**C**) or Rho GTPase cycle and its regulators (**D**). Nominal p-value, normalized enrichment score (NES), and false discovery rates (FDRs) are shown in each plot. n.s., not significant, * *p* < 0.05; ** *p* < 0.01; *** *p* < 0.001.

Since CD40/CD40LG or CD28/CD80/86 signaling promotes cancer progression and chemoresistance via activating the PI3K-AKT and NF-κB pathways in other cancers [38,39,40] and as both pathways are hyperactivated in T-ALL [41,42,43], we then sought to determine whether these pathways function as the downstream cascades of CD40LG or CD28 activation in non-ETP T-ALL cells. To address this, we measured phosphorylation levels of AKT (p-AKT) and p65 (p-p65) following CD40LG or CD28 stimulation. Immunoblotting revealed robust induction of p-AKT and p-p65 induction in MOLT-4 and CEM cells upon activation, whereas Jurkat cells exhibited minimal changes (Figure 4A), indicating constitutive activation of both pathways at baseline in Jurkat cells.

Since the induction of PI3K-AKT signaling was more pronounced than that of the NF-κB pathway following CD40LG/CD28 activation in non-ETP T-ALL cells, we next tested whether CD40LG- or CD28-mediated growth enhancement depends on PI3K-AKT activation. Unexpectedly, compared with controls, Jurkat and MOLT-4 cells stimulated with rhCD40 or hCD28-mAb displayed resistance to buparlisib, a pan-class I PI3K-AKT inhibitor, whereas CEM cells retained similar sensitivity (Figure 4B). These findings indicated that CD40LG or CD28 activation in Jurkat and MOLT-4 cells might drive growth via alternative downstream signaling cascades. To explore this possibility, we performed RNA sequencing (RNA-seq) to profile transcriptional changes in Jurkat and MOLT-4 cells upon CD40LG or CD28 activation. Total mRNA profiles from rhCD40- or hCD28-mAb-treated cells were compared against the respective controls. As gene set enrichment analysis (GSEA) evaluates coordinated expression changes across all measured genes to uncover collective functional trends, we applied GSEA to identify activated pathways. Consistent with prior observations, pathways related to DNA replication and cell-cycle progression including “mitotic spindle”, “DNA replication” and “S phase” were significantly upregulated upon CD40LG or CD28 activation. Additionally, CD40LG activation further enhanced the “regulation of cytoskeleton organization”, a signaling that is implicated in cell migration (Figure 4C). Remarkably, pathways associated with the Rho GTPase cycle and its effectors were identified as the most significantly upregulated signaling modules in both Jurkat and MOLT-4 cells upon CD40LG or CD28 activation (Figure 4D), revealing Rho GTPase signaling as a shared downstream cascade. Likely, the observed resistance of Jurkat and MOLT-4 cells to buparlisib (Figure 4B) is attributed to the CD40LG/CD28-driven upregulation of Rho GTPase signaling.

### 2.4. Blocking Rho GTPase Signaling with Small-Molecule Inhibitors Sensitizes Non-ETP T-ALL Cells to Buparlisib

The Rho GTPase family is a key regulator of cytoskeletal organization, with subfamilies (e.g., Rho, Rac, and Cdc42) orchestrating diverse cellular processes including cell polarity, migration, vesicle trafficking, and cytokinesis [44,45]. Notably, RhoA, RhoC, RhoU (members of the Rho subfamily) and their regulatory factors have been shown to enhance T-ALL cell migration [46,47,48], whereas Rac1 and Rac2 (members of the Rac subfamily) have been implicated in promoting migration and survival of ALL and acute myeloid leukemia cells [49,50]. However, the impact of Rho GTPase signaling on chemoresistance and organ-specific infiltration in non-ETP T-ALL remains incompletely defined.

**Figure 5:**
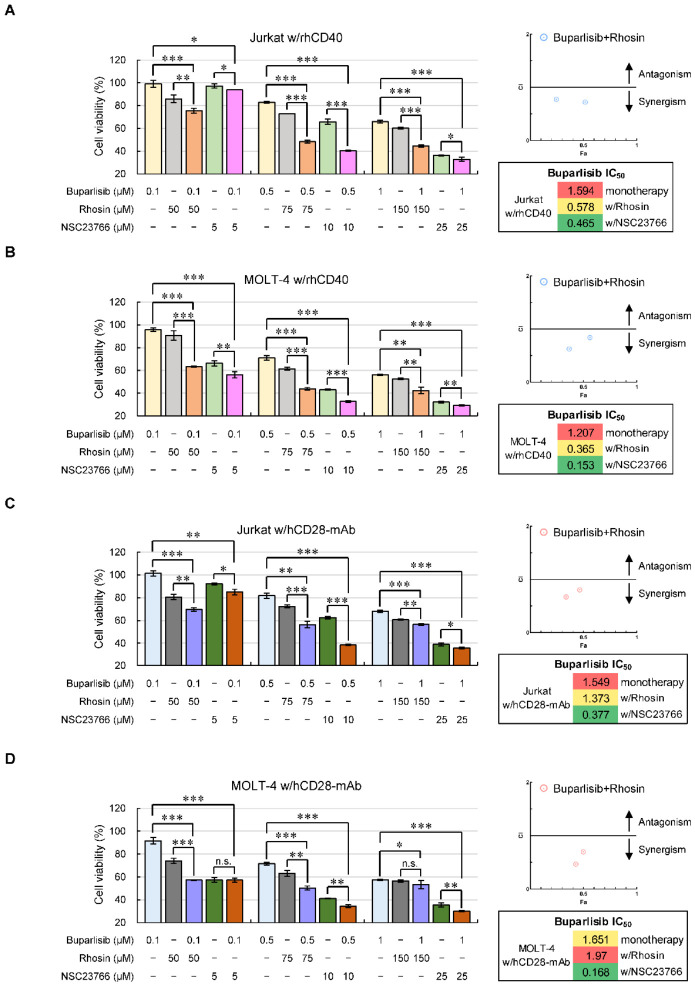
Pharmacological blocking of RhoA or Rac1 induces cytotoxicity and sensitizes resistant leukemic cells to PI3K inhibition. Jurkat (**A**) or MOLT-4 (**B**) cells were treated with buparlisib alone or in combination with Rhosin or NSC23766, in the presence of rhCD40 for 48 h. Jurkat (**C**) or MOLT-4 (**D**) cells were treated with buparlisib alone or in combination with Rhosin or NSC23766, in the presence of hCD28-mAb for 48 h. Cell viability was measured via CellTiter Blue assays, normalized to control. Data is shown as the mean ± SD. Synergic cytotoxic effects of buparlisib and Rhosin were determined by CI using cell viability data. Fa, fraction affected. IC_50_ values of buparlisib alone or in combination are indicated by color intensity, with red representing higher values, green representing lower values, and yellow representing intermediate values. n.s., not significant, * *p* < 0.05; ** *p* < 0.01; *** *p* < 0.001.

In this context, we first evaluated the effects of RhoA and Rac1 inhibitors on Jurkat and MOLT-4 cells following CD40LG or CD28 activation. As anticipated, the RhoA inhibitor Rhosin and Rac1 inhibitor NSC23766 elicited potent cytotoxicity in both CD40LG/CD28-activated cell lines. More importantly, pharmacological inhibition of either signaling remarkably sensitized cells to buparlisib (Figure 5A–D). Further analysis using combination index (CI) plots demonstrated a synergistic cytotoxic effect when Rhosin was combined with low-dose buparlisib in Jurkat and MOLT-4 cells (Figure 5A–D). Although no obvious synergistic effect was observed for the combination of buparlisib and NSC23766, co-treatment with these two agents markedly reduced the IC**_50_** of buparlisib to a minimum value, indicating potent chemo-sensitization (Figure 5A–D). In summary, these results indicated that RhoA and Rac1 signaling are two critical downstream effectors mediating CD40LG/CD28-driven survival in non-ETP T-ALL cells. Inhibition of either pathway not only exerts direct cytotoxicity but also potentiates the anti-leukemia efficacy of buparlisib.

**Figure 6:**
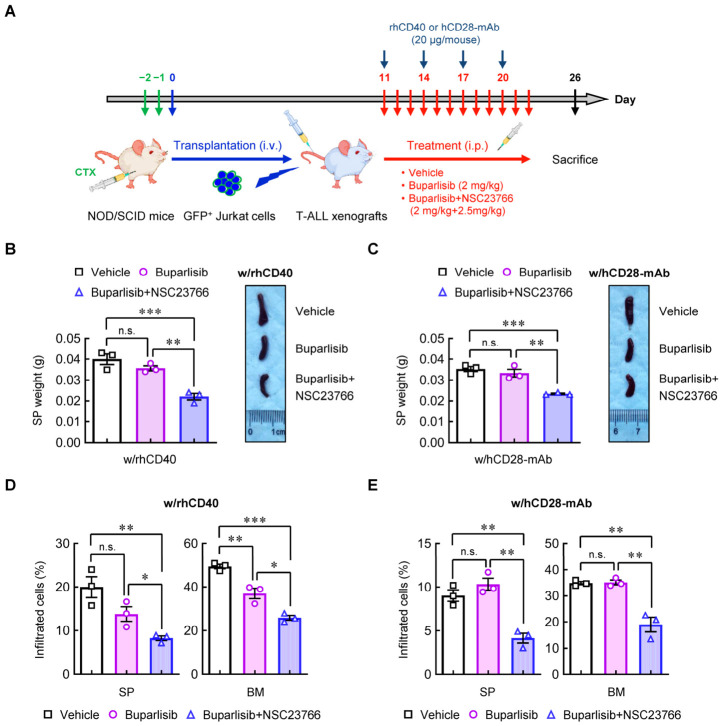
Pharmacological blockade of Rac1 sensitizes resistant leukemic cells to PI3K inhibition in xenografts. (**A**) Experimental design for testing the chemo-sensitizing activity of Rac1 inhibitor NSC23766 for buparlisib in non-ETP T-ALL xenografts. Spleens (SPs) from rhCD40-stimulated (**B**) and hCD28-mAb-stimulated (**C**) xenografts were isolated and measured (left), with representative spleens photographed against a ruler in centimeters (right). GFP^+^ cells from spleens and BMs in rhCD40-stimulated (**D**) and hCD28-mAb-stimulated (**E**) groups were analyzed using flow cytometry. Data are shown as the mean ± SEM. n.s., not significant; * *p* < 0.05; ** *p* < 0.01; *** *p* < 0.001.

Given that Rho GTPases regulate cytoskeletal dynamics and cell motility to promote tissue infiltration, we further explored the therapeutic potential of related interventions. Considering the prominent chemo-sensitizing effect of NSC23766 demonstrated in in vitro assays (Figure 5A–D), we subsequently assessed its efficacy in leukemia xenograft models. Ten days after intravenous injection of Jurkat cells, mice were treated with either buparlisib alone (monotherapy) or a combination of buparlisib and NSC23766 (buparlisib+NSC23766), alongside administration of rhCD40 or hCD28-mAb (Figure 6A). Upon CD40LG or CD28 stimulation, mice receiving the buparlisib + NSC23766 combination exhibited significantly smaller spleens and lower spleen weights than those in the DMSO-treated control group and the buparlisib monotherapy group (Figure 6B,C). This combination regimen also achieved greater suppression of leukemia burden in the spleens and BMs than controls or monotherapy (Figure 6D,E). These results, together with the in vitro data, support the conclusion that Rac1 inhibition potentiates the anti-leukemia effects of buparlisib. Nonetheless, no significant differences were observed between the control and buparlisib monotherapy groups, particularly under CD28 activation (Figure 6C,E), suggesting that blocking the PI3K-AKT pathway alone is insufficient to inhibit T-ALL infiltration into the spleen and BM.

### 2.5. Pharmacological Blockade of Integrin α5β1 Exhibits Minimal Cytotoxicity in Non-ETP T-ALL Cells

Beyond its canonical receptor CD40, CD40LG expressed on T cells also interact with integrin family members (e.g., α5β1, αIIbβ3 and αMβ2) to mediate distinct biological functions [51,52,53,54]. Notably, prior work revealed that CD40LG forms a cis-interaction with integrin α5β1 on the surface of T-ALL cells, thereby suppressing Fas-, TRAIL-, and TNF-α-induced cell death via inhibition of caspase-8 cleavage [55,56,57]. To specifically interrogate this non-canonical role, we employed ATN-161, a small-molecule inhibitor of integrin α5β1, and volociximab, a mAb targeting integrin α5β1, to disrupt the α5β1/CD40LG cis-interaction independent of classical CD40 or CD28 trans-signaling. Unexpectedly, neither agent induced significant cytotoxicity (Supplementary Figure S4A,B), suggesting that ATN-161 and volociximab likely bind to epitopes on integrin α5β1 that are spatially distinct from the CD40LG interaction site, thereby failing to effectively perturb this specific cis-interface.

### 2.6. Elevated CD40LG, CD28, RHOA or RAC2 Expression Correlates with Poor Prognosis in Non-ETP T-ALL Patients

ETP-ALL is a highly aggressive T-ALL subtype with a poorer prognosis than non-ETP T-ALL [4]. However, as the most prevalent T-ALL subtype, non-ETP T-ALL remains plagues by chemoresistance and treatment failure in a subset of patients [6,7]. In this context, identifying diagnostic biomarkers and developing targeted therapies for non-ETP T-ALL patients is of critical importance.

**Figure 7:**
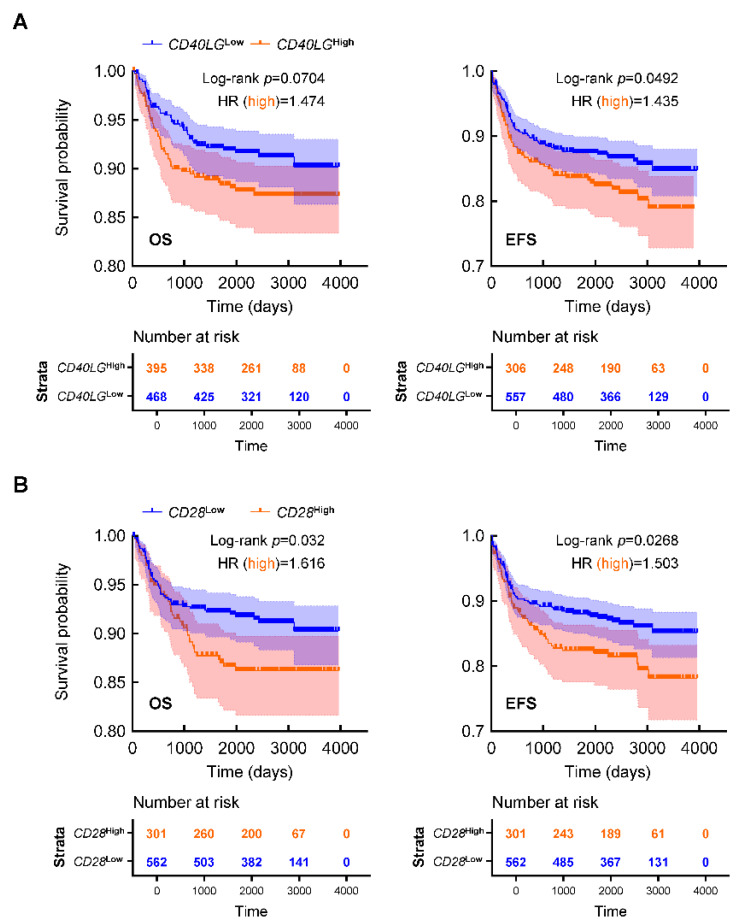
Elevated *CD40LG*/*CD28* expression correlates with poor prognosis in non-ETP T-ALL patients. Kaplan–Meier plots of OS and EFS in non-ETP T-ALL patients (*n* = 863), stratified by high vs. low *CD40LG* expression (**A**) or *CD28* expression (**B**). Log-rank *p* values, HR for high-expression cohorts, and number at risk are indicated.

To determine the clinical relevance of our findings, we next analyzed a publicly available dataset to evaluate the impact of *CD40LG* or *CD28* expression levels on patient survival [58]. As shown in Figure 7A, patients with high expression of *CD40LG* (*CD40LG***^high^**) showed lower overall survival (OS) than those with low expression (*CD40LG***^low^**), although this difference did not reach statistical significance (*p* = 0.0704); however, *CD40LG***^high^** patients exhibited significantly reduced event-free survival (EFS) (*p* = 0.0492). In contrast, *CD28***^high^** patients demonstrated significantly shorter OS (*p* = 0.032) and EFS (*p* = 0.0268) compared with *CD28***^low^** counterparts (Figure 7B). These clinical data indicate that elevated *CD40LG* or *CD28* expression correlates with a more aggressive and progressive phenotype in non-ETP T-ALL, establishing them as adverse prognostic biomarkers. Interestingly, in sharp contrast to non-ETP T-ALL, elevated *CD28* expression in ETP-ALL patients was associated with improved OS and EFS (Supplementary Figure S5A). This paradox may be contextualized by developmental differences: ETP-ALL arises from a very immature hematopoietic stage characterized by the initial migration of hematopoietic stem cells into the thymus [59], whereas CD28 is typically expressed at later stages of T-cell development [25]. Thus, high CD28 expression in ETP-ALL likely reflects a partial shift toward a more differentiated phenotype. Consequently, elevated *CD28* may counteract the stem cell-like properties inherent to ETP-ALL, thereby conferring a more favorable prognosis.

**Figure 8:**
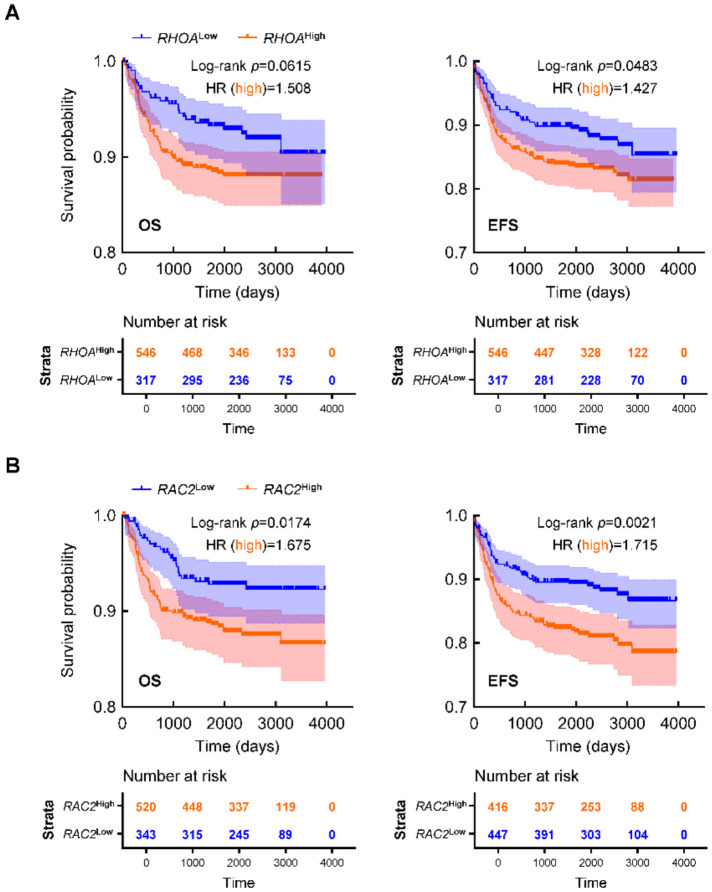
Elevated *RHOA* or *RAC2* expression correlates with unfavorable prognosis in non-ETP T-ALL patients. Kaplan–Meier plots of OS and EFS in non-ETP T-ALL patients (*n* = 863), stratified by high vs. low *RHOA* expression (**A**) or *RAC2* expression (**B**). Log-rank *p* values, HR for high-expression cohorts, and number at risk are indicated.

Next, we explored the role of the Rho GTPase family in non-ETP T-ALL patient survival by individually analyzing correlations between OS/EFS and expression levels of *RHOA* (RhoA), *RHOC* (RhoC), *RAC1* (Rac1), *RAC2* (Rac2), and *RAC3* (Rac3). As shown in Figure 8A, *RHOA***^high^** patients exhibited shorter OS and EFS than *RHOA***^low^** patients. *RAC2***^high^** patients also showed significantly reduced OS and EFS compared with *RAC2***^low^** counterparts (Figure 8B). This result indicates that elevated *RHOA* or *RAC2* expression is associated with an unfavorable prognosis in non-ETP T-ALL. In contrast, *RHOC* or *RAC3* exerted opposing effects on non-ETP T-ALL patient survival (Supplementary Figure S5B,C), whereas *RAC1* expression demonstrated no significant association with survival. These findings not only validate our findings implicating Rho GTPase signaling in the pathogenesis and progression of non-ETP T-ALL but also highlight the distinct functional roles of individual subfamily members, an area that warrants further investigation.

## 3. Discussion

The biological heterogeneity of T-ALL, manifested by immunophenotypic diversity, distinct gene expression profiles, and activation of divergent intracellular signaling cascades, poses significant challenges to the development of effective molecularly targeted and personalized therapies [60]. While non-ETP T-ALL has a more favorable prognosis than ETP-ALL, a subset of non-ETP T-ALL cases still progress to R/R T-ALL [6,7]. Thus, identifying novel therapeutic targets that drive non-ETP T-ALL progression has become an urgent priority.

CD40LG and CD28 are essential costimulatory molecules critical for T-cell activation and proliferation [23,24,61,62,63]. Our prior work demonstrated that both molecules are indispensable for the growth and survival of non-ETP T-ALL cells, as their complete knockdown induces rapid cell death [32]. However, the precise molecular mechanisms underlying CD40LG- or CD28-mediated signaling remain poorly defined. In the current study, we found that CD40LG or CD28 activation not only promotes non-ETP T-ALL cell growth by accelerating cell-cycle progression but also enhances pro-migratory properties to facilitate homing to the BM microenvironment. Notably, CD40LG upregulates CXCR4 to enhance BM homing, aligning with its established role in BM infiltration, cell migration and metastasis [21,22,32]. In contrast, CD28 may rely on alternative chemokines for adhesion and migration, highlighting pathway-specific mechanisms.

To elucidate the downstream pathways mediated by CD40LG or CD28, we integrated prior literature insights with RNA-seq data. We observed that both the PI3K-AKT and NF-κB pathways are activated in MOLT-4 and CEM cells following CD40LG or CD28 stimulation; however, activation of these pathways was not evident in Jurkat cells, likely due to their pre-existing constitutive activation caused by mutations in *PTEN* as reported previously [33,34,35]. Importantly, RNA-seq analyses revealed the Rho GTPase signaling pathway as a pivotal downstream effector of CD40LG or CD28 activation. Indeed, pharmacological blockade of RhoA or Rac1 not only induced potent anti-leukemic cytotoxicity but also sensitized resistant cells to buparlisib. Subsequent in vivo experiments further validated the robust chemo-sensitizing potential of Rac1 inhibition for buparlisib, indicating that Rho GTPase signaling maintains PI3K-independent survival signaling in CD40LG/CD28-activated cells. Of note, all non-ETP T-ALL cell lines used in this study carry *TP53* alterations, which recapitulate the genetic features of high-risk, R/R T-ALL. Therefore, our findings not only uncover a novel molecular mechanism but also identify CD40LG/CD28 signaling as a promising therapeutic target for this molecularly distinct patient subgroup with extremely poor prognosis.

Lastly, we provided clinical evidence that elevated expression of *CD40LG*, *CD28*, *RHOA* or *RAC2* correlates with poor prognosis in non-ETP T-ALL, supporting their potential as novel biomarkers for clinical risk stratification. In patients with high CD40LG expression, OS showed a non-significant declining trend, whereas EFS was significantly impaired. Such consistent trends suggest that the marginal OS difference is biologically meaningful, and further validation in larger prospective cohorts is therefore warranted. Similarly, while *RAC1* expression showed no significant prognostic correlation, elevated *RAC2* expression was strongly associated with inferior clinical outcomes. These findings imply that the Rac1 inhibitor NSC23766 may exert off-target therapeutic effects via concurrent suppression of Rac2 signaling in non-ETP T-ALL cells. Intriguingly, *RHOC* or *RAC3* exhibited opposing prognostic effects on non-ETP T-ALL survival, highlighting the functional complexity and divergent roles of individual Rho GTPase subfamily members, a topic requiring further investigation.

## 4. Materials and Methods

### 4.1. Cell Lines and Culture

Human T-ALL cell lines Jurkat E6-1 and CCRF-CEM were purchased from BNCC (Beijing, China) and authenticated by short tandem repeat (STR) profiling; human T-ALL cell line MOLT-4 was purchased from Cobioer Biosciences (Nanjing, China) and authenticated by STR profiling. The human bone marrow stromal cell line HS-5 was purchased from BNCC (Beijing, China) and authenticated by STR profiling at CinoAsia Institute (Shanghai, China). All cell lines were confirmed mycoplasma-free and cultured in RPMI1640 or DMEM (as appropriate for each line) supplemented with 10% fetal bovine serum (FBS) and 100 U.I./mL penicillin–streptomycin. Cells were incubated at 37 °C in a humidified atmosphere with 5% CO_2_. GFP-positive (GFP^+^) Jurkat cells used for xenograft experiments were generated by lentiviral transduction using a non-targeting GFP-expressing vector, as previously described [32].

### 4.2. Immunoblotting and Antibodies

Cells were pretreated with rhCD40 (Novoprotein, Suzhou, China; 0.5μg/mL) or hCD28-mAb (Novoprotein, 5μg/mL) for specific durations, followed by immunoblotting as previously described [64]. Semiquantitative analysis of immunoblots was performed using ImageJ v1.51j8 software (NIH, Bethesda, MD, USA), with target protein expression normalized to β-Actin. Antibodies used included: β-Actin (66009-1-Ig) from Proteintech (Rosemont, IL, USA); P65 (#4764), p-P65 (#3033), AKT (#9272), p-AKT (#9271), and secondary antibodies from Cell Signaling Technology; and CXCR4 (CD184, #555976) from BD Pharmingen (San Diego, CA, USA).

### 4.3. Cell-Cycle and Apoptosis Assays

Cells were pretreated with vehicle control, rhCD40 (0.5 μg/mL) and/or hCD28-mAb (5 μg/mL) for 48 h (h). For cell-cycle analysis, cells were collected, washed twice with ice-cold PBS, and resuspended in PI/RNase Staining Buffer (BD Biosciences, San Jose, CA, USA) for 15 min at room temperature in the dark. For apoptosis assays, cell collection and washing steps were performed identically, followed by staining with the PE Annexin V Apoptosis Detection Kit (BD Biosciences) according to the manufacturer’s instructions. Trypan Blue exclusion assay verified that cell viability remained above 90% in all groups before staining. All stained cells were analyzed using a CytoFLEX flow cytometer (Beckman Coulter, Brea, CA, USA), and raw data were processed with FlowJo^TM^ v10.8.1 software (BD Life Sciences, Ashland, OR, USA).

### 4.4. Surface Expression Analysis

For the detection of CD40LG and CD28, cells were pretreated with rhCD40 (0.5 μg/mL) or hCD28-mAb (5 μg/mL) for 48 h. For CXCR4 detection, cells were pretreated with vehicle control, rhCD40 (0.5 μg/mL) or hCD28-mAb (5 μg/mL) for 16 h. After treatment, cells were collected and washed twice with ice-cold PBS. Surface immunofluorescence staining was then performed using anti-human-CD40LG (CD154), anti-human-CD28, or anti-human-CXCR4 (CD184) antibodies (BD Pharmingen) for 30 min at 4 °C in the dark. Following another two washes with ice-cold PBS, samples were analyzed on a CytoFLEX flow cytometer, and data were processed using FlowJo^TM^ v10.8.1 software.

### 4.5. Cell Adhesion Assay

Leukemic cells were pre-stained with the membrane dye PKH26 (Sigma, St. Luis, MO, USA) according to the manufacturer’s protocol, followed by treatment with vehicle control, rhCD40 (0.5 μg/mL) or hCD28-mAb (5 μg/mL) for 16 h. Stained cells were seeded onto the pre-established monolayer of HS-5 cells and incubated for 1h at 37 °C in 5% CO_2_. Adherent cells were quantified by measuring PKH26 fluorescence intensity using a Cytation 1 multifunctional imaging reader (Biotek, Winooski, VT, USA).

### 4.6. Cell Migration and Chemotaxis Assays

Leukemic cells were pre-stained with PKH26 followed by treatment with vehicle control, rhCD40 (0.5 μg/mL) or hCD28-mAb (5 μg/mL) for 16 h. Cells were then resuspended in FBS-free RPMI1640 and added to the upper chambers of 8 μm pore polycarbonate Transwell inserts (Corning, Kennebunk, ME, USA) in 24-well plates. Lower chambers contained culture medium with confluent HS-5 monolayers or complete medium supplemented with recombinant human CXCL12 (rhCXCL12, R&D Systems, Minneapolis, MN, USA; 10 ng/mL). Plates were incubated for 4 h at 37 °C in 5% CO_2_, and migrated cells in lower chambers were quantified by measuring PKH26 fluorescence intensity.

### 4.7. Cell Viability Assay and Determination of CI and IC_50_

All compounds were purchased from MedChemExpress (Monmouth Junction, NJ, USA). For single-agent treatment experiments, leukemic cells were exposed to different doses of buparlisib (#HY-70063), Rhosin (#HY-12646A), NSC23766 (#HY-15723), ATN-161 (#HY-13535) or volociximab (#HY-P99333) for 48 h. For combination treatments, cells were treated with buparlisib alone or in combination with Rhosin or NSC23766, in the presence of rhCD40 (0.5 μg/mL) or hCD28-mAb (5 μg/mL) for 48 h. Viability was assessed via fluorometric quantification using CellTiter-Blue^®^ reagent (Promega, Madison, WI, USA). For cell growth assay, viable cells were evaluated by manual counting as previously described [65,66].

The synergic cytotoxicity of buparlisib combined with Rhosin or NSC23766 was evaluated using the CI method [67]. CI values were calculated and plotted using CompuSyn v1.0.1 software (ComboSyn, Inc., Paramus, NJ, USA) as previously described [68]. IC_50_ values of buparlisib alone or in combination were calculated using GraphPad Prism 9 v9.5.1 software (GraphPad Software, San Diego, CA, USA).

### 4.8. RNA-Seq Profiling and GSEA Analysis

Total RNAs were extracted from Jurkat and MOLT-4 cells treated with vehicle control, rhCD40 (0.5 μg/mL) or hCD28-mAb (5 μg/mL) (*n* = 2/group). RNA libraries were constructed and sequenced on an Illumina NovaSeq 6000 platform (Jiayin Biotechnology Co., Shanghai, China). Reads were aligned to the human reference genome (GRCh38) using STAR v2.5.2b software (Cold Spring Harbor Laboratory, Cold Spring Harbor, NY, USA) [69] and quantified with Kallisto v0.48.0 software (California Institute of Technology, Pasadena, CA, USA) [70]. GSEA was conducted using the clusterProfiler R package (v4.6.2) [71]. Gene sets were obtained from Molecular Signature Database (MSigDB) via the R package msigdbr (v7.5.1). Nominal *p* values, normalized enrichment scores (NESs), and false discovery rates (FDRs) were calculated using default parameters.

### 4.9. Tumor Xenografts and Treatment

Female NOD/SCID mice (6 weeks old, 18–20g) were purchased from Charles River Laboratories (Beijing, China) and housed under specific pathogen-free (SFP) barrier conditions at the Institute of Medical Biology (IMB). All animal procedures were approved by the Institutional Animal Care and Use Committee of IMB. Mice were pretreated with intraperitoneal (i.p.) injections of cyclophosphamide (CTX) at 100 mg/kg once daily for two consecutive days, followed by a single intravenous (i.v.) injection of GFP^+^ Jurkat cells (2 × 10^6^ cells/mouse, *n* = 18) via the tail vein. Ten days after transplantation, mice were randomized into two groups: rhCD40 stimulated (*n* = 9) and hCD28-mAb stimulated (*n* = 9); mice in each group received i.p. administrations of rhCD40 (20 μg/mouse) or hCD28-mAb (20 μg/mouse) every 3 days for a total of 4 injections starting on day 11. Concurrently, all mice were subjected to daily i.p. administrations starting on day 11 for a total of 12 injections, with the following regimens: DMSO (vehicle control), buparlisib monotherapy (2 mg/kg), or the combination of buparlisib (2 mg/kg) and NSC23766 (2.5 mg/kg). Mice were humanely euthanized 4 days after the final treatment dose. Spleens and BMs were collected, and leukemic burden was assessed by quantifying the percentage of GFP^+^ cells (Supplementary Figure S6).

### 4.10. Clinical Data Sources and Analyses

A public clinical dataset of T-ALL patients, comprising 863 non-ETP T-ALL cases and 110 ETP-ALL cases, was obtained from the Synapse platform (accession number: syn54032669) [58]. This dataset contains processed gene expression profiles and survival data. Optimal cutpoints for stratifying patients into high- and low-expression cohorts were determined using the surv_cutpoint function in the survminer R package (v0.5.0), which identifies thresholds maximizing the log-rank statistic for survival differences. Kaplan–Meier survival curves were plotted to visualize OS and EFS differences between cohorts using GraphPad Prism 9 v9.5.1 software. Survival differences were assessed by the log-rank test, and hazard ratios (HRs) with 95% confidence intervals were calculated to quantify the association between gene expression and prognosis.

### 4.11. Statistical Analysis

Data are presented as mean ± standard deviation (SD) or standard error of mean (SEM). Differences between two groups were assessed by unpaired Student *t* tests; statistical significance was defined as * *p* < 0.05 and ** *p* < 0.01. All experiments were repeated at least three times and performed in duplicate or triplicate.

## 5. Conclusions

This study uncovers a leukemia-promoting role for CD40LG- or CD28-mediated signaling in non-ETP T-ALL, with elevated expression of these molecules correlating with an unfavorable prognosis. Mechanistically, CD40LG activation enhances cell adhesion and promotes migration by upregulating CXCR4. Furthermore, we identified, for the first time, that the Rho GTPase signaling pathway acts as a pivotal downstream effector of CD40LG or CD28 activation in non-ETP T-ALL cells. Targeting this pathway or its key subfamily members (e.g., RhoA, Rac1) may represent a promising therapeutic strategy to overcome chemoresistance, thus offering a novel avenue for intervention in non-ETP T-ALL.

## Data Availability

The published article includes all data generated/analyzed for this study. Other relevant data are available from the corresponding author upon request.

## Supplementary Materials

The following supporting information can be downloaded at https://www.mdpi.com/article/10.3390/ijms27125306/s1.
