# Supplementary material for: CD40LG/CD28-Mediated Rho GTPase Signaling Drives Survival and Chemoresistance in Non-ETP T-ALL
Source: Int J Mol Sci. 2026 Jun 11;27(12):5306. doi: 10.3390/ijms27125306 (PMC13299955; doi:10.3390/ijms27125306)
Supplement: Supplementary file 1 [file ijms-27-05306-s001.zip › ijms-4284340-supplementary.pdf]

Figure S1

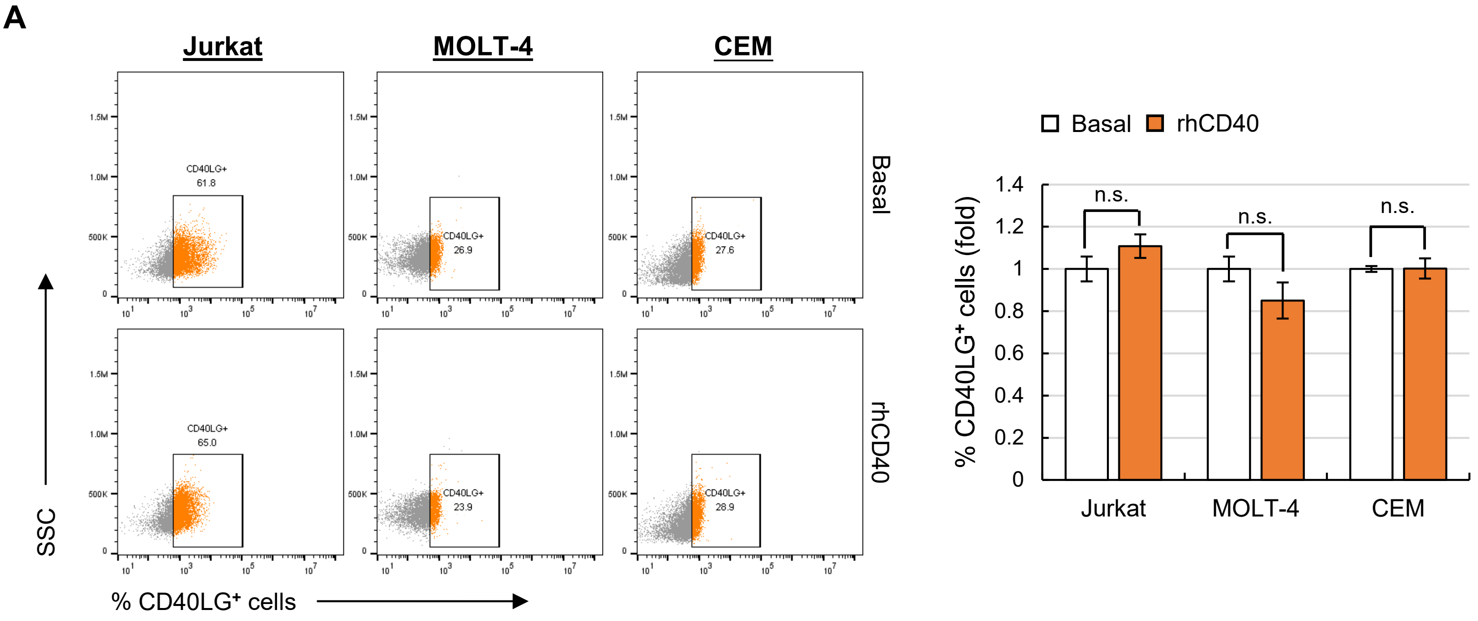

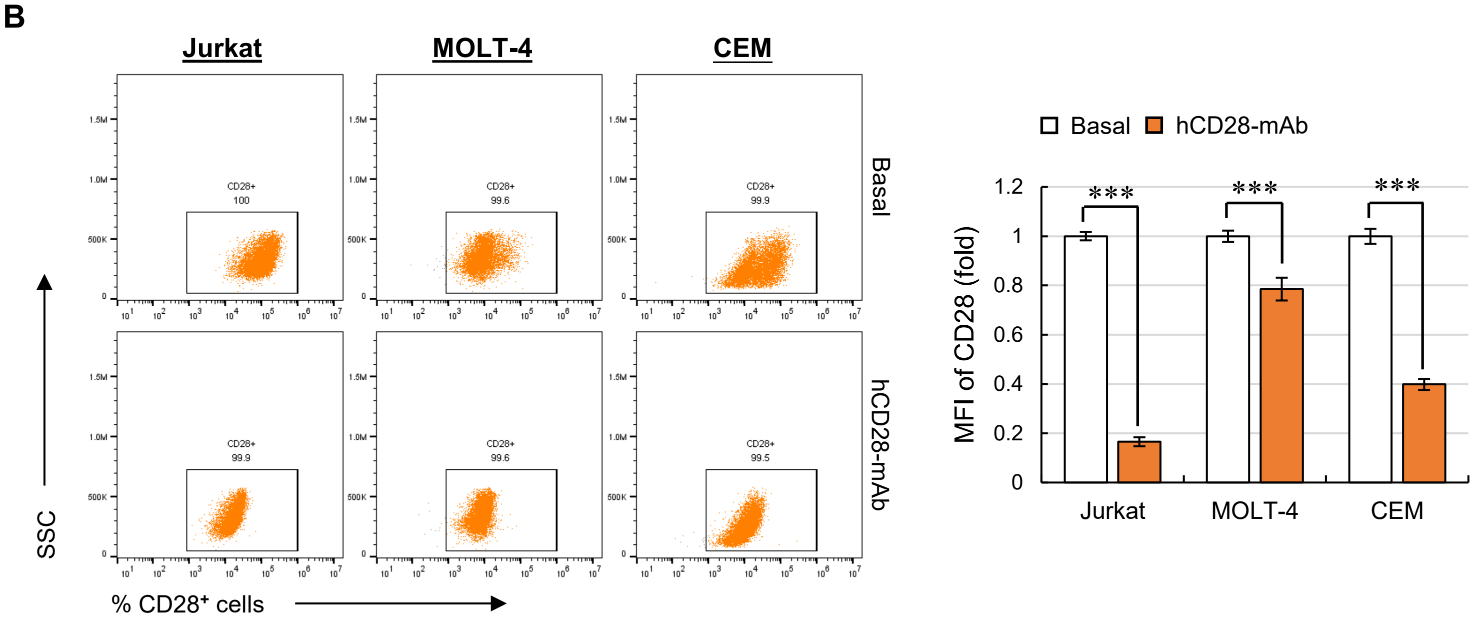

Figure S1. Surface expression of CD40LG and CD28 in non-ETP T-ALL cells under basal and stimulated conditions. (A) Representative flow cytometric plots showing surface CD40LG expression in cells under basal conditions and following rhCD40 stimulation (left). The percentage of CD40LG⁺ cells is normalized to the basal group (right). **(B)** Representative flow cytometric plots for surface CD28 expression under basal conditions and after hCD28-mAb stimulation (left). The mean fluorescence intensity (MFI) of CD28 is normalized to the basal group (right). Data are shown as mean ± SD. n.s., not significant, ****p* <0.001.

Figure S2

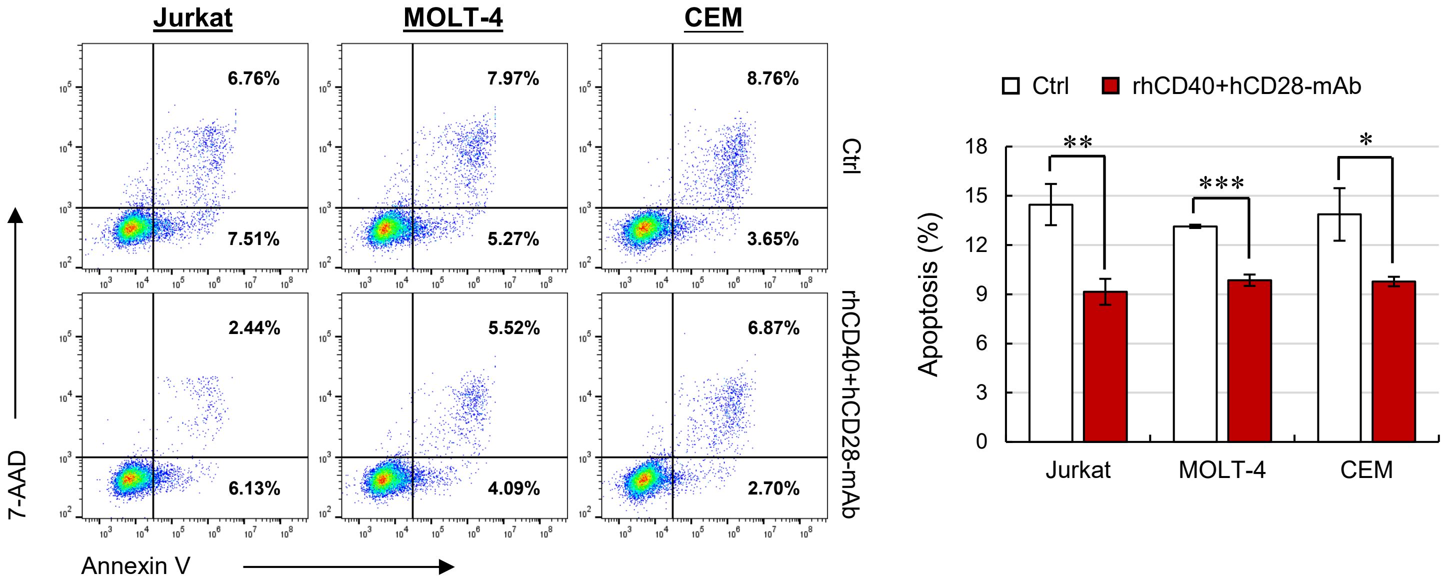

Figure S2. Activation of both CD40LG and CD28 signaling promotes non-ETP T-ALL survival. Representative apoptosis (left) and quantification of Annexin V+ apoptotic cells (right) for Jurkat, MOLT-4 and CEM cells treated with vehicle control or rhCD40+hCD28-mAb. Data are shown as mean ± SD. n.s., not significant, **p* <0.05; ***p* <0.01; ****p* <0.001.

Figure S3

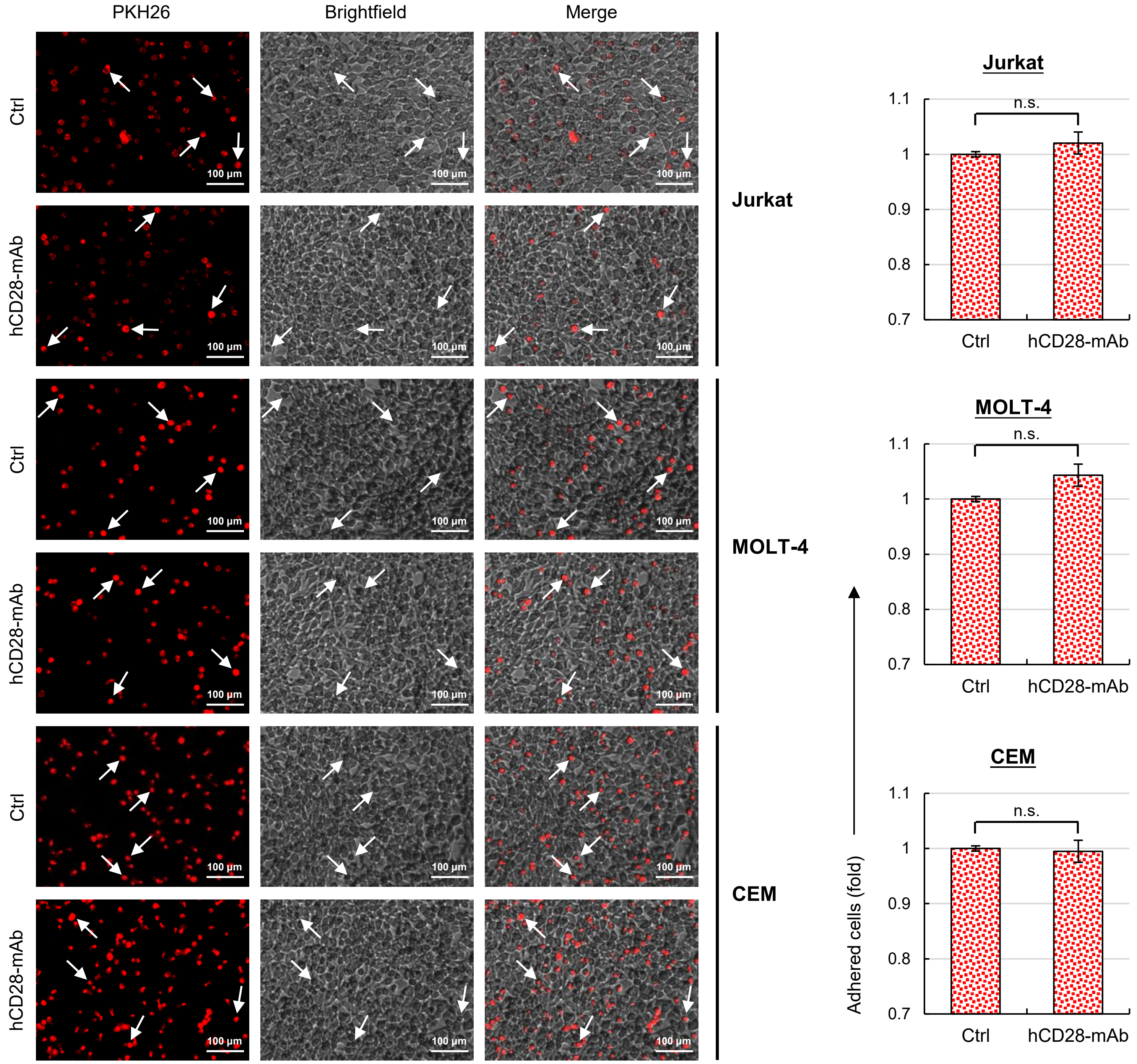

Figure S3. The effect of CD28 simulation on the adhesion of non-ETP ALL cells to HS-5 BMSCs. Representative microscopy images of adherent Jurkat, MOLT-4 and CEM cells treated with vehicle control or hCD28-mAb, with arrows indicating leukemic cells adhering to BMSCs (left, scale bar, 100 μm). PKH26 fluorescence intensity in hCD28-mAb-treated cells normalized to control (right). Data are shown as mean ± SD. n.s., not significant.

Figure S4

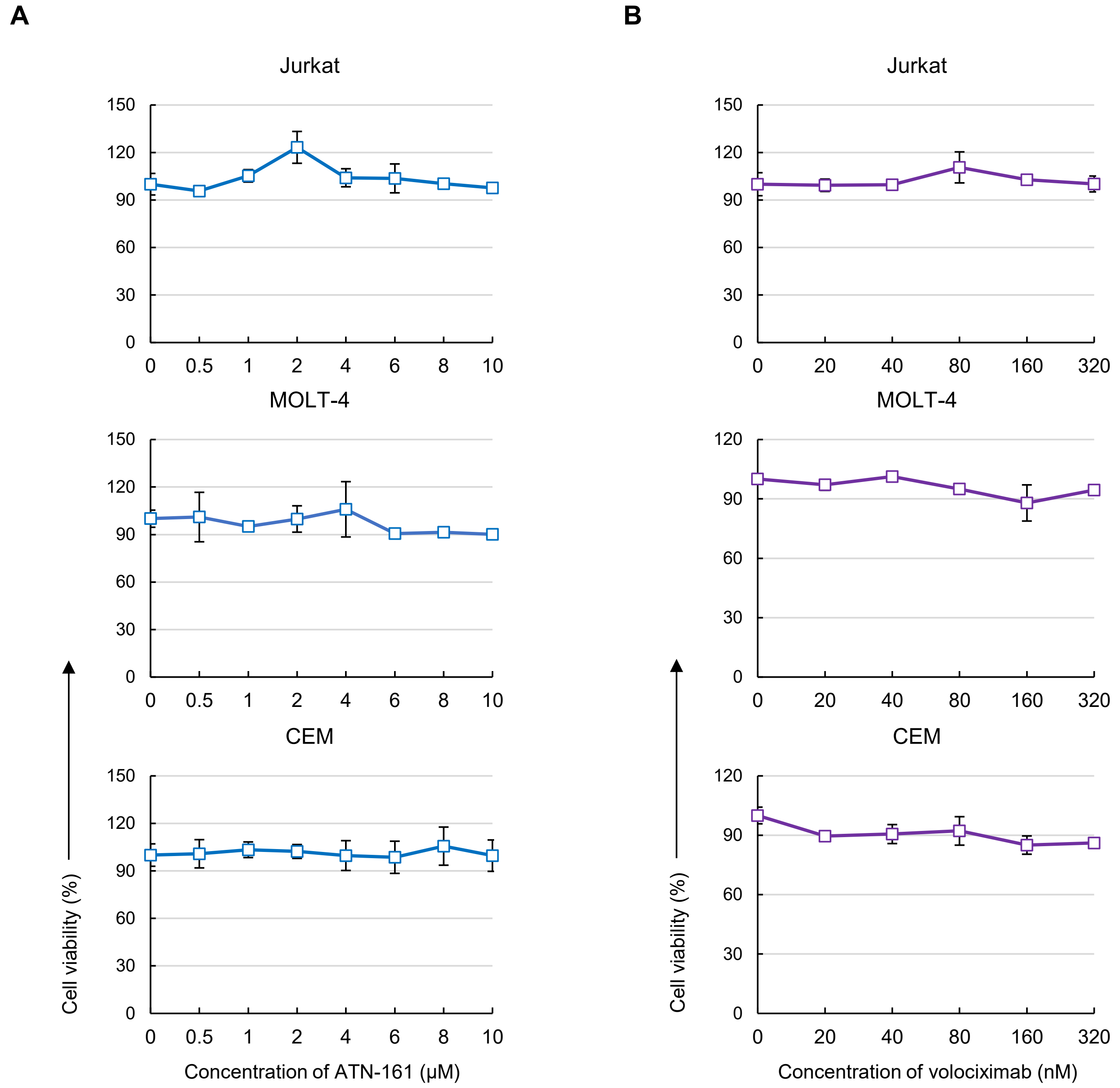

Figure S4. The cytotoxicity of ATN-161 and volociximab against non-ETP ALL cells. Cells were treated with varying doses of integrin α5β1 antagonist ATN-161 **(A)** or volociximab targeting integrin α5β1 **(B)** for 48h. Cell viability was measured via CellTiter Blue assays, normalized to control. Data are shown as the mean ± SD.

Figure S5

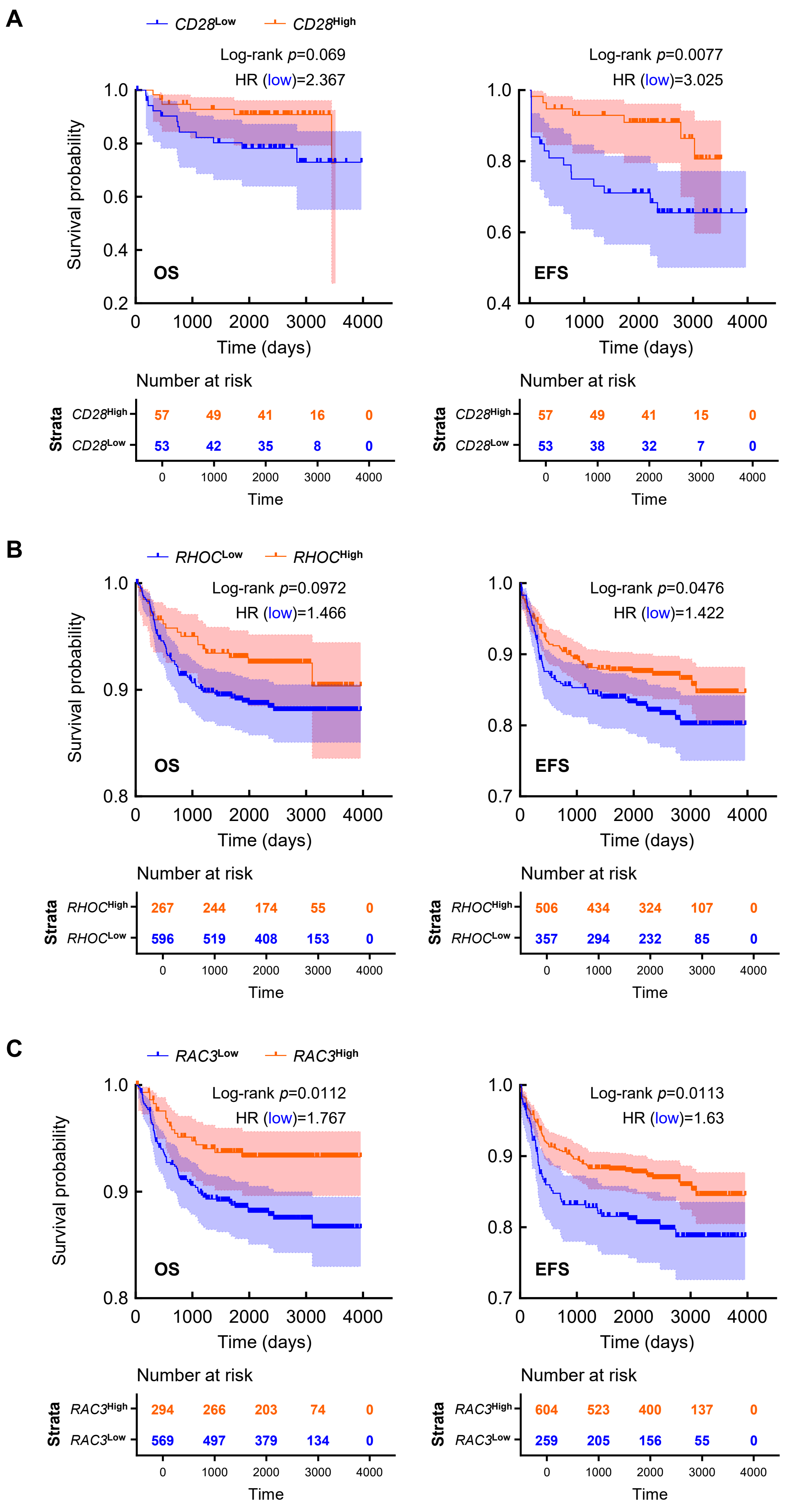
A
CD28Low
CD28High
Log-rank p=0.069
HR (low)=2.367
Survival probability
OS
Time (days)
Log-rank p=0.0077
HR (low)=3.025
EFS
Time (days)
Number at risk
Strata
CD28High 57 49 41 16 0
CD28Low 53 42 35 8 0
Time
Number at risk
Strata
CD28High 57 49 41 15 0
CD28Low 53 38 32 7 0
Time
B
RHOCLow
RHOCHigh
Log-rank p=0.0972
HR (low)=1.466
Survival probability
OS
Time (days)
Log-rank p=0.0476
HR (low)=1.422
EFS
Time (days)
Number at risk
Strata
RHOCHigh 267 244 174 55 0
RHOCLow 596 519 408 153 0
Time
Number at risk
Strata
RHOCHigh 506 434 324 107 0
RHOCLow 357 294 232 85 0
Time
C
RAC3Low
RAC3High
Log-rank p=0.0112
HR (low)=1.767
Survival probability
OS
Time (days)
Log-rank p=0.0113
HR (low)=1.63
EFS
Time (days)
Number at risk
Strata
RAC3High 294 266 203 74 0
RAC3Low 569 497 379 134 0
Time
Number at risk
Strata
RAC3High 604 523 400 137 0
RAC3Low 259 205 156 55 0
Time

Figure S5. Association of *CD28*, *RHOC* or *RAC3* expression with different subtypes of T-ALL patients. (A) Kaplan-Meier plots of OS and EFS in ETP-ALL patients (n=110), stratified by high vs. low *CD28* expression. Kaplan-Meier plots of OS and EFS in non-ETP ALL patients (n=863), stratified by high vs. low *RHOC* expression **(B)** or *RAC3* expression **(C)**. Log-rank *p* values, HR for low-expression cohorts, and number at risk are indicated.

Figure S6

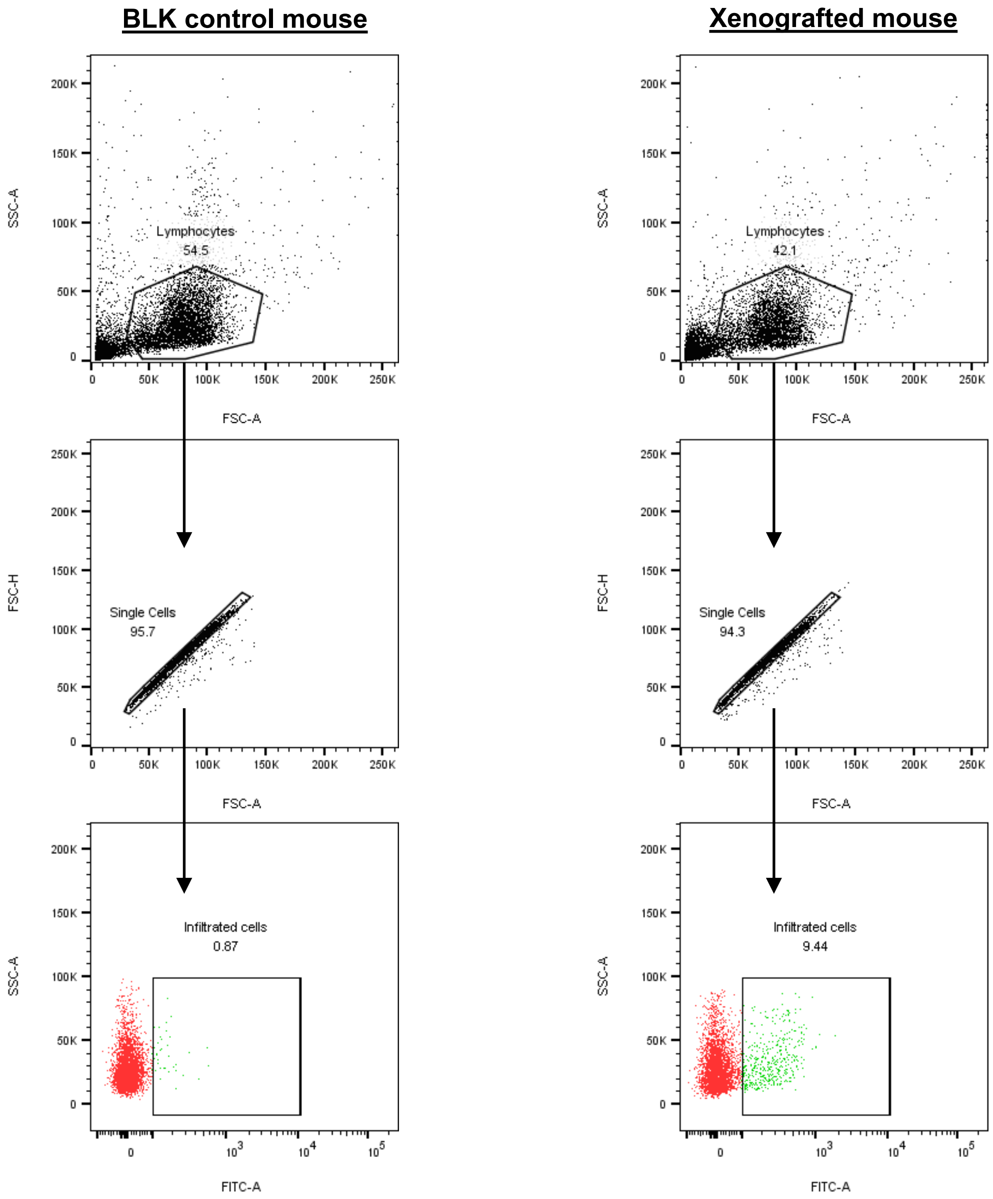

Figure S6. Gating strategy for flow cytometric analysis of infiltrated GFP$^+$ cells in mouse tissues. Representative flow cytometry plots showing the sequential gating strategy used to identify infiltrated human cells in the spleen of a blank control (BLK) mouse (left) and a GFP$^+$ Jurkat-xenografted mouse (right). Cells were first gated by FSC-A and SSC-A to identify the lymphocyte population (top panels). Single-cell events were then selected using FSC-A vs. FSC-H to exclude doublets (middle panels). The gate for infiltrated GFP$^+$ cells in the FITC (GFP) channel was defined based on the fluorescence background observed in the BLK control mouse (bottom panels). The percentage of events within each gate is indicated.
